# Clinical performance during 48 months of two current glass ionomer restorative systems with coatings: a randomized clinical trial in the field

**DOI:** 10.1186/s13063-016-1339-8

**Published:** 2016-05-08

**Authors:** Thomas Klinke, Amro Daboul, Anita Turek, Roland Frankenberger, Reinhard Hickel, Reiner Biffar

**Affiliations:** Polyclinic of Prosthodontics and Biomaterials, Greifswald University, Rotgerberstr. 8, Greifswald, 17475 Germany; Conservative Dentistry Department, Philipps University of Marburg, Marburg, Germany; Policlinics for Restorative Dentistry and Periodontology, University of Munich, Munich, Germany

**Keywords:** Practice-based network, Dental restoration, Permanent, Glass ionomer, Multi-center study

## Abstract

**Background:**

This study was carried out as a prospective clinical field study with the aim of evaluating the clinical performance of Equia Fil® with a nanofilled resin coating and the conventional Fuji IX GP® fast with an LC coating according to the World Dental Federation (FDI) restoration material evaluation criteria.

**Methods:**

The clinical performance of Equia Fil® and Fuji IX GP® fast was evaluated on permanent posterior teeth of 643 adult patients aged between 20 to 80 years old in randomly selected clinics across Germany. Occlusal cavities in posterior permanent teeth were restored with Equia Fil® with a nanofilled, light-cured resin coating (*n* = 515) and Fuji IX GP® fast with an LC coating (*n* = 486). Direct clinical assessment as well as photographic assessment and assessment of stone casts of the restorations were made at 1 year, 2 years, 3 years, and 4 years.

**Results:**

In 4 years, a total of 1001 fillings from both materials were placed by 111 dentists in 643 patients. Random slope models showed that the Equia filling system had overall lower odds of obtaining a delta event (material needs replacement) in comparison to Fuji IX GP® fast with an LC coating within all models. In both materials, filling size/surface was the most important component affecting the clinical performance of the materials. When measuring the odds of obtaining a delta event (material needs replacement), the odds ratios jumped to approximately 43 and 296 times for class II (two surfaces) and class II mesial-occlusal-distal (three surfaces) respectively in comparison to class I fillings.

**Conclusion:**

Both materials showed similar good overall performance in class I cavities; however, when including numbers from both class I and II fillings, the Equia system with a nanofilled resin coating showed better overall performance with fewer failures in all the follow-up intervals. Nonetheless, the percentage of unsatisfactory to poor fillings according to the FDI criteria was relatively high in two-surface class II fillings and higher in three-surface class II fillings for both materials.

**Trial registration:**

Deutsches Register Klinischer Studien (German Clinical Trials Register): DRKS00004220. (www.germanctr.de). Registration date: 6 Sept 2012.

**Electronic supplementary material:**

The online version of this article (doi:10.1186/s13063-016-1339-8) contains supplementary material, which is available to authorized users.

## Background

Since their development in the 1970s, glass ionomer cements (GICs) have been widely used as a restorative material in no-load bearing surfaces, mainly in class III and V cavities. The principal advantages of GICs are as follows: direct chemical adhesion to tooth substance, good compressive strength, the ability to remineralize dental tissues through fluoride release, and ease of use under different clinical settings. Nonetheless, a GIC is considered a semi-permanent restoration material for class I and class II cavities in permanent teeth (in countries with high economic status). The use of a conventional GIC as a permanent restoration material is often questioned because of its poor tensile and flexural strengths which may result in a higher rate of early fractures and also occlusal wear compared to other filling materials. The relatively low fracture resistance of a traditional GIC in comparison with other filling materials can be attributed to its low fracture toughness [1].

Developers of the new generation of GICs have tried to overcome this disadvantage by introducing a fast-setting reinforced glass ionomer, which should provide protection in the early maturation phase and improve strength and surface hardness [2].

Nonetheless, several in vitro studies on high-strength conventional GICs showed an inferior wear resistance compared to composite resin and a higher wear resistance compared to resin-modified GIC [3–6]. However, reported results from these studies differed significantly among the different wear mechanisms. In a 6-year retrospective clinical study evaluating 116 class II cavities filled with fast-setting conventional GIC (Fuji IX GP® fast), no failures were observed in the first 1.5 years. Survival of fillings dropped to 93 % after 3.5 years and to 60 % after 6 years [7]. In another retrospective clinical trial, Burke et al. [8] reported a survival rate of 98 % after 2 years for conventional GIC (Fuji IX GP® fast) fillings in class I and class II cavities. They found that the main reason for replacement was fracture of the fillings. In a systemic review on the longevity of fillings in posterior teeth, Hickel et al. [9] reported that fractures in the GIC fillings caused an annual failure rate between 1.4 to 14 %, which is higher than that of amalgam and composites.

In recent years, an encapsulated glass ionomer for which the manufacturer claims high mechanical properties has been marketed [10]. The fast-setting high-viscosity GIC coated with a nanofilled resin (Equia Fil®, GC Corp., Tokyo, Japan) is supposed to have an increased wear resistance and is advertised as a replacement for amalgam and composite fillings in class I and II cavities within the manufacturer’s recommended cavity isthmus width. The nanofilled resin coating seals surface defects of the underlying GIC material and protects against abrasive wear and early material fractures. This is of particular importance in the initial days of GIC filling placement until it has matured and its peak strength is reached [11].

Considering that previous studies showed that the wear resistance of high-strength conventional GIC is inferior to that of composite and amalgam, evidence is needed to determine whether the application of a nanofilled resin as a coating for GIC fillings in class I and II cavities would increase their wear and fracture resistance.

Different studies were carried out to show the effect of applying a coating material on the wear and fracture resistance of GIC fillings in class I and II fillings. Although many in vitro studies showed that applying a coating material increased the wear resistance of GIC and even made it comparable to the wear resistance of composite resin [12–14], plus with higher flexural strength [15], many clinical reports and trials were indeterminate or conflicting. While some studies showed high annual failure rates for GIC fillings in class II cavities [16, 17], other studies reported excellent clinical outcomes for both class I and II GIC fillings [18–21].

Nonetheless, different results on the clinical performance of coated GIC fillings can be attributed to factors like operator, cavity design, type of study, evaluation period, and criteria for failure or success.

Moreover, although there were studies that compared GIC fillings with and without coatings, there are no studies comparing GIC fillings with different coatings. In addition, most of the previous studies were either retrospective studies or short-term prospective clinical trials on GICs under ideal university environment conditions. It is therefore the aim of this trial in the field to evaluate, under “real-world” conditions, the clinical performance of Equia Fil® and Fuji IX GP® fast. Fillings placed in adult subjects, who represent the daily patients, by private dental practices across Germany will be examined and evaluated at 1-year intervals for 4 years.

## Methods

A double blind, randomized, prospective clinical field study was primarily designed to assess the clinical performance of two brands of GIC promoted as an alternative filling material in posterior teeth. The recruitment process of dentists and patients from private dental clinics was evaluated and characterized according to the socio-economic status and geographic districts of residence in Germany. A full description of the recruitment process is described in detail elsewhere [22].

To ensure statistical power, the minimal representative sample size was based on the required number of fillings to evaluate both materials (*n* = 440 for each group). The homogeneity of the participating clinics was guaranteed by the recruitment and the criterion that only one or two filling(s) will be placed for each patient. Therefore, an exponential maximum likelihood test of equality with a *p* = 0.05 two-sided significance level will have 90 % power to detect the difference between the Group 1 (Equia fillings) exponential parameter, a γ1 of 20 % 0.021 (corresponding to a proportion of 30 % after 60 months), and the Group 2 (LC coated Fuji IX) exponential parameter, a γ2 of 0.026 (corresponding to a proportion of 20 % after 60 months), with a constant hazard ratio of 0.021/0.026 = 0.75, assuming an accrual period of 12 months, a maximum follow-up time of 60 months, and a common exponential dropout rate of 1 %.

### Recruitment

The dental practitioners’ recruitment process began in September 2009 and was completed in July 2011. A total of 3194 private dental clinics were invited, of which 53.6 % (*n* = 1712) refused to participate, 36.3 % (*n* = 1159) did not respond to the invitation, and 10.1 % (*n* = 323) agreed to participate. Of the 323 clinics that agreed to participate, only 144 clinics (44.6 %) (4.5 % of invited clinics, mean 7.1 % of invited clinics in all regions) participated in the lectures held in their cities and signed the participation agreement.

In the participating dental clinics, qualified patients were asked to participate in the study only when a dental filling was indicated on posterior teeth. A qualified patient for the study had to fulfill the study’s inclusion criteria (adult subjects with full dentition, no partial or full dentures, and at least three zones with occlusal contact on natural teeth in the posterior region). Exclusion criteria were patients with craniomandibular disorder, patients with teeth out of occlusion, and patients who refused to sign the consent form.

Patients who agreed to participate had to sign an informed consent of agreement according to Good Clinical Practice (GCP) and the Declaration of Helsinki. Each participant was assigned a unique pseudonym in the dental practice, and the unique pseudonym key remained in the list of patients at the dental practice.

The clinical trial was approved by the ethical commission at Greifswald University (number: BB 33/09).

### Calibration

A lecture held in each participating city educated and familiarized the participating dentists with the internationally approved directives for clinical trials according to GCP [23]. Participating dentists were also briefly informed on the specific processing methodology of Good Manufacturing Practices (GMP) [24] and Good Epidemiological Practice (GEP). The study operating procedure was then discussed, and the precise clinical trial conditions were given in printed form to each participant. At the end of the lecture, a certificate of participation was handed out to each participating dentist. Later on, each participating dental practice received a package by mail with ten de-identified and relabeled filling capsules with their coatings (five relabeled boxes of the Equia Fil® system as label A and five relabeled boxes of LC coated Fuji IX GP® fast as label B), so neither the dental practitioner nor the patient knew in this blinded design which material was used.

Each finished filling was given a pseudonym consisting of four digital fields: (1) practitioner ID, (2) patient pseudonym, (3) cavity class, and (4) material label.

Finally, all pseudonymized information that was collected from participating dentists and patients was stored and monitored by a special committee. The Data Safety and Monitoring Committee (DSMC) based at Munich University guaranteed the abidance of randomization and quality assurance of the data acquisition and database.

### Application

The cavities included in this study were limited to single-surface occlusal cavities and two-surface cavities (mesial-occlusal, MO or distal-occlusal, DO) with or without buccal/lingual extension. Multi-surface (more than two surfaces) cavities were not included, except for the case of unexpected cavity extension to a multi-surface filling during treatment.

After caries excavation a “triple tray” impression of the cavity was made using a light-body vinyl polysiloxane impression material (Exafast NDS, GC Corp., GC America Inc., Alsip, IL, USA). Stone models were constructed from the impression and used later to analyze cavity design preparation and size. The manufacturer’s indications for both materials limited the cavity size to small occlusal cavities with an isthmus size of 50 % of the buccal-oral intercuspal distance. The cavity was cleaned, water rinsed, and conditioned with 20 % polyacrylic acid and 3 % aluminum chloride solution using Cavity Conditioner® (GC Corp., Tokyo, Japan) for 20 s and finally rinsed with water. The cavity surface was lightly dried with cotton pellets and the automatically mixed study material (10 s) was slowly injected into the cavity. After 45 s the filling material was formed into shape, and after the total setting time of 2.5 min the restoration was trimmed and adapted using high-speed, water-cooled fine diamond burs (125 μm). Finally the surface was sealed with the light-cured system-dependent coating.

A triple tray impression was taken using the light-body impression material (Exafast NDS, GC Corp., GC America Inc.) after the treatment.

### Evaluation and follow-up

A record file of all the inserted fillings was sent to the study center in Greifswald. The fillings were evaluated by three calibrated, external, certified examiners. The calibration and certification of the external examiners was done through individual lectures and tested with the Internet tool “e-calib” (see Hickel et al. [25]). During a defined week (12 months +/- after Filling placement date) an examiner visited the participating dental clinics, where all treated patients were invited to join the follow-up weeks before. During the follow-up examination, the fillings were examined using 4.5× magnifying glasses (Zeiss, Germany). Intraoral digital photographs and triple tray replicas of the relevant arches were taken using a light-body vinyl impression material (Exafast NDS, GC Corp., GC America Inc.) to aid in the evaluation and comparison of the fillings and detect any small reductions or fractures in each filling between the follow-up examinations. The clinical records enclosed the pseudonymized patients’ data, the treated tooth (location and number of the tooth), filling surface(s), and the FDI evaluation criteria described by Hickel et al. [25]. Neither the patients nor the dentists and follow-up examiners knew which filling material had been placed. The evaluation criteria were organized into three groups: aesthetic parameters (four criteria), functional parameters (six criteria), and biological parameters (six criteria). Each criterion can be expressed with five scores, three for acceptable and two for non-acceptable (one for repairable [score 4] and one for replacement [score 5]). The selected FDI criteria for direct restorations in this study were surface luster (A1), fracture of material and retention (B5), marginal adaptation (B6), occlusal contour and wear (B7), approximal anatomical form (B8), patient’s view (B10), postoperative sensitivity and tooth vitality (C11), recurrence of caries, erosion, abfraction (C12), and tooth integrity (C13). The date of failure (if any), reason for failure (e.g., chipping, fracture), and the date of last attendance (if the patient missed the appointment) were recorded.

### Statistical methods

Statistical analyses were performed with Stata/MP software, release 12.1 (Stata Corporation, College Station, TX, USA). The change in clinical criteria over time was estimated with logistic mixed models by using the gllamm procedure [26]. Mixed models use all available data, properly account for correlation between repeated measurements in dentists, patients, and in teeth, and appropriately handle missing data if the missing at random assumption is met.

Clinically poor and clinically unsatisfactory fillings (criteria B5, B6, B8, B10, C11, C12, or C13 according to Hickel et al. [25]) were defined as cases. Correspondingly, clinically sufficient fillings (or better) were defined as non-cases. Clinically poor fillings were handled by the last observation carried forward method, which is clinically justified for poor, but not for unsatisfactory, fillings.

Because baseline values were not assessed, the treatment effect estimates the difference in change over the first half year, whereas the treatment * time effect estimates the difference in change between treatment groups after the first half year. By dropping the measurements of the first half year, the factor “treatment” becomes thoroughly interpretable, namely as the difference in change in treatment from baseline to the measurement after the first year. Although the baseline values are unknown, it is justified to assume equal baseline values over treatment groups because of the large sample size. Thus, the treatment * time effect estimates the difference in change between treatment groups after the first half year. For fixed effects, a *P* value < 0.05 was considered statistically significant.

As recommended [26], we built simple to complex models. The first model assumes independence of each observation, which is clearly violated. The second model assumes independence of each filling, which is violated if more than one filling per patient was placed (random intercept for filling). The third to seventh models account properly for correlation between repeated measurements in patients (random intercept for patient). The eighth model accounts additionally for repeated measurements in dentists (random intercept for dentist). The fourth to eighth models adjust for age and sex.

## Results

A total of 1006 fillings from both materials were placed by 111 dentists in 643 patients. Five fillings were excluded before follow-ups because they had not fulfilled the inclusion criteria (no occlusal surface). Thus, the number of total fillings was *n* = 1001: 486 fillings with Fuji IX GP® fast with an LC coating and 515 fillings with the Equia Fil® system. Out of 1001 fillings, 219 fillings were excluded because the patients failed to appear for the follow-up exam (details on patients who are missing and lost to follow-up are available in Additional file 1: Additional tables.). A total of 782 fillings (384 made from Fuji IX GP fast® with an LC coating and 398 fillings made with the Equia Fil® system) were examined in the follow-ups and included in the analysis, including fillings (1) within the manufacturer indications, (2) within and without the manufacturer indications, and (3) with missing data on indication. Only 503 fillings (245 Fuji IX GP® fast with an LC coating and 258 fillings with the Equia Fil® system) were identified as within the manufacturer indications.

The overall clinical performance score for both materials (*n* = 782) in 510 patients with 1713 follow-up exams is shown in Table 1.

**Table 1:** Overall clinical performance score (number and percentage) for both materials (*n* = 782) in 510 patients with 1713 follow-up exams

	1 year		2 years		3 years		4 years	
Material	GP fast	Equia	GP fast	Equia	GP fast	Equia	GP fast	Equia
Score 1 (clinically excellent)	37 (13)	53 (16)	30 (13)	44 (16)	22 (12)	20 (12)	10 (8)	18 (15)
Score 2 (clinically good)	137 (46)	150 (46)	108 (46)	151 (54)	79 (43)	77 (47)	49 (41)	52 (44)
Score 3 (clinically sufficient)	71 (24)	78 (24)	47 (20)	53 (19)	33 (18)	26 (16)	13 (11)	15 (13)
Score 4 (clinically unsatisfactory)	40 (13)	32 (10)	27 (12)	12 (4)	13 (7)	13 (8)	8 (7)	2 (2)
Score 5 (clinically poor)	10 (3)	10 (3)	22 (9)	18 (6)	35 (19)	28 (17)	38 (32)	32 (27)
Total	295	323	234	278	182	164	118	119

In class I fillings (*n* = 312), five fillings had score 5 (clinically poor, filling needs replacement) in category B5 (fracture) in the span of 4 years (one Fuji IX GP® fast with an LC coating and four Equia Fil® system fillings).

In class II fillings (MO or DO) (*n* = 436), 47 events of score 5 (clinically poor, filling needs replacement) in categories B5 (fracture) and B8 (loss of approximal contact) were observed in the span of 4 years (27 Fuji IX GP® fast with an LC coating and 20 Equia Fil® system fillings).

In three surface class II fillings (mesial-occlusal-distal, MOD, *n* = 34), four events of score 5 (clinically poor, filling needs replacement) in categories B5 (fracture) and B8 (loss of approximal contact) were observed in the span of 4 years (three Fuji IX GP® fast filling with an LC coating and one Equia Fil® system filling).

The number and percentage of fractured fillings from observed fillings in all filling classes in distributed follow-up checks are shown in Table 2. The approximal anatomical form evaluation in distributed follow-up checks for class II and class II MOD fillings is shown in Table 3.

**Table 2:** First part of the description of the overall clinical performance score: number and percentage (in parentheses) of fractured fillings in distributed follow-up checks

Class	Material	Fillings	Classification score; *n* = observations	1 year	2 years	3 years	4 years	Poor total
I	GP fast	146	*n*	106	91	65	38	
			1 (clinically excellent)/	106 (100)	91 (100)	62 (95)	38 (100)	
			2 (clinically good)/					
			3 (clinically sufficient)					
			4 (clinically unsatisfactory)	0 (0)	0 (0)	2 (3)	0 (0)	
			5 (clinically poor)	0 (0)	0(0)	1 (2)	0 (0)	1
	Equia Fil	166	*n*	126	120	63	55	
			1 (clinically excellent)/	124 (98)	119 (99)	63 (100)	55 (100)	
			2 (clinically good)/					
			3 (clinically sufficient)					
			4 (clinically unsatisfactory)	1 (1)	0 (0)	0 (0)	0 (0)	
			5 (clinically poor)	1 (1)	1 (1)	0 (0)	0 (0)	2
II	GP fast	225	*n*	176	125	90	50	
			1 (clinically excellent)/	168 (95)	108 (86)	80 (89)	44 (88)	
			2 (clinically good)/					
			3 (clinically sufficient)					
			4 (clinically unsatisfactory)	7 (4)	10 (8)	5 (6)	3 (6)	
			5 (clinically poor)	1 (1)	7 (6)	5 (6)	3 (6)	16
	Equia Fil	211	*n*	175	138	81	35	
			1 (clinically excellent)/	169 (97)	132 (96)	71 (88)	34 (97)	
			2 (clinically good)/					
			3 (clinically sufficient)					
			4 (clinically unsatisfactory)	4 (2)	5 (4)	6 (7)	1 (3)	
			5 (clinically poor)	2 (1)	1 (1)	4 (5)	0 (0)	7
II MOD	GP fast	13	*n*	10	10	5	2	
			1 (clinically excellent)/	9 (90)	9 (90)	3 (60)	1 (50)	
			2 (clinically good)/					
			3 (clinically sufficient)					
			4 (clinically unsatisfactory)	1 (10)	1 (10)	0 (0)	0 (0)	
			5 (clinically poor)	0 (0)	0 (0)	2 (40)	1 (50)	3
	Equia Fil	21	*n*	18	11	4	2	
			1 (clinically excellent)/	18 (100)	10 (91)	3 (75)	2 (100)	
			2 (clinically good)/					
			3 (clinically sufficient)					
			4 (clinically unsatisfactory)	0 (0)	0 (0)	1 (25)	0 (0)	
			5 (clinically poor)	0 (0)	1 (9)	0 (0)	0 (0)	1

**Table 3:** Second part of the description of the overall clinical performance score: number and percentage of two and three-surface fillings that lost approximal contact in distributed follow-up checks

Class	Material	Fillings	Classification score; *n* = observations	1 year	2 years	3 years	4 years	Poor total
II	GP fast	225	*n*	160	111	76	42	
			1 (clinically excellent)/	135 (84)	96 (86)	67 (88)	36 (86)	
			2 (clinically good)/					
			3 (clinically sufficient)					
			4 (clinically unsatisfactory)	21 (13)	11 (10)	8 (11)	4 (10)	
			5 (clinically poor)	4 (2)	4 (4)	1 (1)	2 (5)	11
	Equia Fil	211	*n*	160	116	71	28	
			1 (clinically excellent)/	141 (88)	106 (91)	60 (85)	26 (93)	
			2 (clinically good)/					
			3 (clinically sufficient)					
			4 (clinically unsatisfactory)	16 (10)	5 (4)	7 (10)	1 (4)	
			5 (clinically poor)	3 (2)	5 (4)	4 (6)	1 (4)	13
II MOD	GP fast	13	*n*	10	10	3	2	
			1 (clinically excellent)/	8 (80)	10 (100)	3 (100)	2 (100)	
			2 (clinically good)/					
			3 (clinically sufficient)					
			4 (clinically unsatisfactory)	1 (10)	0 (0)	0 (0)	0 (0)	
			5 (clinically poor)	1 (10)	0 (0)	0 (0)	0 (0)	1
	Equia Fil	21	*n*	16	9	5	2	
			1 (clinically excellent)/	9 (57)	7 (78)	2 (40)	2 (100)	
			2 (clinically good)/					
			3 (clinically sufficient)					
			4 (clinically unsatisfactory)	5 (31)	2 (22)	1 (20)	0 (0)	
			5 (clinically poor)	2 (12)	0 (0)	2 (40)	0 (0)	4

In the random slope models, the Equia Fil® filling system showed an overall lower odds ratio (OR) in obtaining a score 5 (clinically poor, filling needs replacement) in comparison to Fuji IX GP® fast with an LC coating within all models. (The OR values were 0.43, 0.20, 0.18, 0.19, 0.20, 0.25, 0.29, 0.36 in models 1–8 respectively.)

In both materials, the number of surfaces (class I, II, and class II MOD) was the most important component affecting the clinical performance of the materials. When measuring the odds of obtaining a score 5, the odds ratios jumped to approximately 42 and 296 times for class II and class II MOD respectively in comparison to class I fillings.

Within the random slope models, both materials showed a steady increase in obtaining a score 5 when the time effect component was included. Moving from simpler to more complex models involving the components jaw, treated tooth and location, cavity class, number of fillings per patient, and dentist effects, no significant change in the odds ratios was found when the filling was in the upper or lower jaw. Relatively higher odds ratios for score 5 (replacement) were associated with second and third molars in comparison with premolars, using the first premolar as a reference, but only in class II fillings. Additionally, significantly higher odds ratios for a score 5 were noticed in class II fillings when the tooth component was included (OR 90.1). Odds ratios for a score 5 were relatively higher in both materials when the new filling replaced an old existing filling in the patient, but only after 3 years (OR 6.45 in GP fast and 8.99 in Equia). The dentist effect (specialized versus general practitioner) did not seem to have any significant impact on odds ratios of failure in both materials. Tables 4 and 6 show the effect of all components (time, jaw, treated tooth and location, cavity class, number of fillings per patient, and dentist effects) on both materials, including fillings (1) within the manufacturer indications, (2) not within the manufacturer indications, and (3) with missing data on indication, with and without the first half-year observations.

**Table 4:** Random intercept models adjusted for fillings, patients, and dentists for all fillings (within and outside the manufacturer’s indications) without 0 year: excluding observations in the first half year (total = 1713 observations)

	Model 1	Model 2	Model 3	Model 4	Model 5	Model 6	Model 7	Model 8
				Adjusted for age and sex	Adjusted for age and sex	Adjusted for age and sex	Adjusted for age and sex	Adjusted for age and sex
*P* value for treatment	0.127	0.123	0.104	0.125	0.125	0.124	0.142	0.194
*P* value for treatment * time	0.857	1.000	0.996	0.981	1.000	0.827	0.877	0.892
Joint *P* value for treatment and treatment * time	0.005	0.055	0.070	0.087	0.092	0.122	0.130	0.191
Fixed part: odds ratios								
Material Equia (reference: GP fast)	0.63 (0.35–1.14)	0.38 (0.11–1.30)	0.34 (0.09–1.25)	0.36 (0.10–1.32)	0.37 (0.10–1.32)	0.38 (0.11–1.30)	0.40 (0.12–1.36)	0.45 (0.14–1.50)
Material GP fast: time effect								
Reference: 1 year	1	1	1	1	1	1	1	1
2 years	1.48 (1.25–1.75)	2.05 (1.51–2.78)	2.05 (1.52–2.77)	2.06 (1.52–2.78)	2.04 (1.51–2.76)	2.03 (1.51–2.74)	2.04 (1.51–2.75)	2.04 (1.52–2.75)
3 years	2.18 (1.56–3.05)	4.21 (2.29–7.74)	4.21 (2.30–7.68)	4.24 (2.32–7.75)	4.18 (2.29–7.62)	4.14 (2.28–7.51)	4.16 (2.29–7.54)	4.18 (2.30–7.58)
4 years	3.23 (1.96–5.32)	8.63 (3.45–21.5)	8.62 (3.49–21.3)	8.74 (3.54–21.6)	8.54 (3.47–21.0)	8.43 (3.45–20.6)	8.48 (3.47–20.7)	8.53 (3.49–20.9)
Material Equia: time effect								
Reference: 1 year	1	1	1	1	1	1	1	1
2 years	1.45 (1.24–1.68)	2.05 (1.49–2.82)	2.05 (1.49–2.83)	2.05 (1.49–2.82)	2.04 (1.49–2.76)	2.13 (1.55–2.94)	2.11 (1.53–2.90)	2.10 (1.53–2.89)
3 years	2.09 (1.55–2.84)	4.21 (2.22–7.97)	4.21 (2.22–8.00)	4.20 (2.21–7.97)	4.18 (2.21–7.91)	4.55 (2.40–8.63)	4.44 (2.35–8.39)	4.43 (2.35–8.33)
4 years	3.03 (1.92–4.78)	8.63 (3.31–22.5)	8.65 (3.30–22.6)	8.90 (3.29–22.5)	8.54 (3.28–22.2)	9.71 (3.72–25.3)	9.36 (3.61–24.3)	9.31 (3.61–24.0)
Upper jaw (ref: lower jaw)	---	---	---	---	0.91 (0.38–2.15)	---	0.71 (0.32–1.57)	0.69 (0.32–1.50)
Tooth type (reference: 4)								
5	---	---	---	---	5.08 (1.30–19.9)	---	4.52 (1.32–15.5)	4.42 (1.32–14.8)
6	---	---	---	---	2.43 (0.64–9.26)	---	3.51 (1.01–12.1)	3.11 (0.92–10.6)
7	---	---	---	---	1.45 (0.38–5.52)	---	5.64 (1.55–20.5)	4.59 (1.30–16.3)
8	---	---	---	---	1.19 (0.09–15.8)	---	8.85 (0.72–108)	10.9 (0.93–127)
Class (reference: class I)								
II	---	---	---	---	---	42.6 (13.9–131)	60.0 (18.1–199)	51.1 (15.7–167)
II MOD	---	---	---	---	---	296 (43.5–2013)	318 (46.4–2179)	279 (40.9–1899)
Random part:								
Filling: intercept	---	4.64 (3.37–5.93)	2.51 (1.71–3.31)	2.54 (1.73–3.34)	2.53 (1.71–3.34)	2.27 (1.48–3.05)	2.24 (1.45–3.02)	2.18 (1.41–2.94)
Patient: intercept	---	---	3.44 (2.51–4.37)	3.37 (2.44–4.29)	3.29 (2.38–4.21)	2.87 (2.04–3.69)	2.76 (1.94–3.57)	2.31 (1.48–3.14)
Dentist: intercept	---	---	---	---	---	---	---	1.62 (0.92–2.32)

*interaction effect

**Fig. 1:**
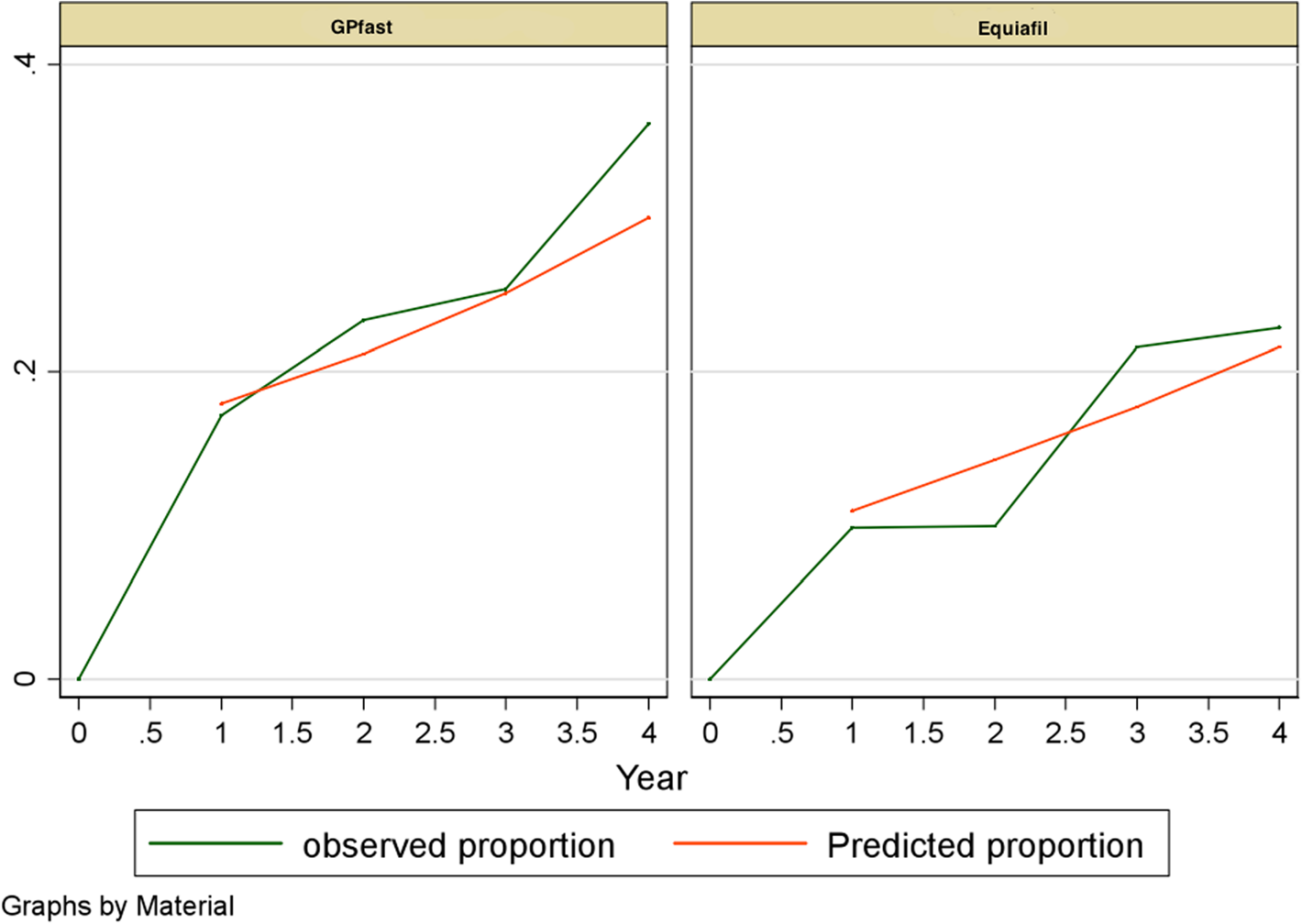
Predicted proportion in a random intercept model adjusted for fillings. Odds of failure for GIC fillings (Fuji IX GP® fast and Equia Fil®) within the manufacturer’s indications

**Fig. 2:**
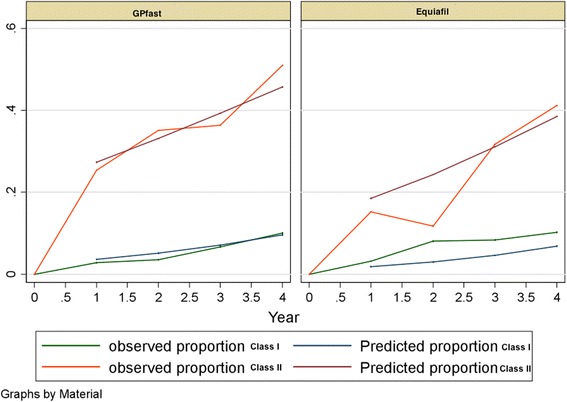
Predicted proportion in a random intercept model adjusted for patients and filling surface. Odds of failure for class I and class II GIC fillings (Fuji IX GP® fast and Equia Fil®)

The effects of all components on fillings only placed within the manufacturer’s indications with and without the first half-year observations are shown in Tables 5 and 7. Observed and predicted odds proportions for both materials are shown in Fig. 1. Observed and predicted odds proportions for both materials in Classes I and II are shown in Fig. 2.

**Table 5:** Random intercept models adjusted for fillings, patients, and dentists for all fillings (only within the manufacturer’s indications) without 0 year: excluding observations in the first half year (total = 1141 observations)

	Model 1	Model 2	Model 3	Model 4	Model 5	Model 6	Model 7	Model 8
		(Fig. 1)					(Fig. 2)	
				Adjusted for age and sex	Adjusted for age and sex	Adjusted for age and sex	Adjusted for age and sex	Adjusted for age and sex
*P* value for treatment	0.027	0.036	0.028	0.033	0.038	0.069	0.097	0.166
*P* value for treatment * time	0.550	0.667	0.613	0.628	0.653	0.515	0.595	0.579
Joint *P* value for treatment and treatment * time	<0.001	0.021	0.027	0.033	0.037	0.112	0.144	0.284
Fixed part: odds ratios								
Material Equia (reference: GP fast)	0.43 (0.21–0.91)	0.20 (0.04–0.90)	0.18 (0.04–0.83)	0.19 (0.04–0.87)	0.20 (0.04–0.91)	0.25 (0.06–1.11)	0.29 (0.07–1.25)	0.36 (0.09–1.52)
Material GP fast: time effect								
Reference: 1 year	1	1	1	1	1	1	1	1
2 years	1.37 (1.14–1.64)	1.86 (1.31–2.65)	1.85 (1.30–2.62)	1.86 (1.31–2.64)	1.86 (1.31–2.64)	1.85 (1.31–2.63)	1.86 (1.31–2.64)	1.86 (1.31–2.63)
3 years	1.86 (1.30–2.68)	3.46 (1.71–7.03)	3.41 (1.69–6.88)	3.45 (1.71–6.96)	3.44 (1.70–6.95)	3.44 (1.71–6.93)	3.45 (1.71–6.95)	3.44 (1.71–6.92)
4 years	2.55 (1.47–4.39)	6.45 (2.23–18.6)	6.30 (2.20–18.1)	6.40 (2.23–18.4)	6.38 (2.22–18.3)	6.38 (2.23–18.2)	6.40 (2.24–18.3)	6.39 (2.24–18.2)
Material Equia: time effect								
Reference: 1 year	1	1	1	1	1	1	1	1
2 years	1.49 (1.20–1.85)	2.08 (1.41–3.06)	2.10 (1.43–3.11)	2.10 (1.43–3.10)	2.08 (1.42–3.07)	2.19 (1.49–3.24)	2.13 (1.45–3.13)	2.14 (1.46–3.13)
3 years	2.22 (1.44–3.42)	4.32 (1.99–9.38)	4.43 (2.03–9.66)	4.43 (2.03–9.64)	4.34 (2.00–9.41)	4.82 (2.21–10.5)	4.53 (2.10–9.81)	4.57 (2.12–9.83)
4 years	3.30 (1.72–6.31)	8.99 (2.81–28.7)	9.32 (2.90–30.0)	9.31 (2.90–29.9)	9.04 (2.83–28.8)	10.6 (3.28–34.0)	9.65 (3.03–30.7)	9.76 (3.09–30.8)
Upper jaw (ref: lower jaw)	---	---	---	---	1.01 (0.36–2.85)	---	0.82 (0.31–2.18)	0.74 (0.29–1.89)
Tooth type (reference: 4)								
5	---	---	---	---	3.70 (0.83–16.5)	---	5.04 (1.24–20.4)	4.53 (1.17–17.5)
6	---	---	---	---	2.35 (0.50–10.9)	---	5.73 (1.30–25.2)	4.55 (1.09–19.1)
7	---	---	---	---	1.94 (0.42–8.98)	---	14.6 (2.91–72.9)	11.1 (2.36–52.0)
8	---	---	---	---	1.27 (0.04–38.6)	---	36.5 (1.34–996)	38.0 (1.58–913)
Class (reference: class I)								
Class II	---	---	---	---	---	42.1 (11.1–161)	90.1 (19.8–410)	78.4 (17.7–348)
Random part:								
Filling: intercept	---	4.25 (2.91–5.59)	2.40 (1.44–3.36)	2.42 (1.45–3.40)	2.42 (1.44–3.41)	2.42 (1.45–3.39)	2.36 (1.39–3.32)	2.34 (1.39–3.29)
Patient: intercept	---	---	3.08 (2.03–4.13)	3.05 (2.00–4.10)	2.96 (1.92–4.01)	2.50 (1.50–3.49)	2.32 (1.34–3.31)	1.44 (0.12–2.75)
Dentist: intercept	---	---	---	---	---	---	---	1.76 (0.98–2.55)

*interaction effect

In Tables 4 and 5 both the treatment effect and the interaction treatment * time can be interpreted, while in Tables 6 and 7 the treatment effect cannot be interpreted because of the absence of baseline values. The difference between both materials is limited to the interaction term with time.

**Table 6:** Random intercept models adjusted for fillings, patients, and dentists for all fillings (within and outside the manufacturer’s indications) with 0 year: including observations in the first half year (total = 2495 observations)

	Model 1	Model 2	Model 3	Model 4	Model 5	Model 6	Model 7	Model 8
				Adjusted for age and sex	Adjusted for age and sex	Adjusted for age and sex	Adjusted for age and sex	Adjusted for age and sex
*P* value for treatment	0.167	0.255	0.209	0.244	0.262	0.244	0.285	0.397
*P* value for treatment * time	0.837	0.541	0.553	0.536	0.533	0.522	0.686	0.639
Joint *P* value for treatment and treatment * time	0.008	0.069	0.062	0.075	0.085	0.138	0.145	0.208
Fixed part: odds ratios								
Material Equia (reference: GP fast)	0.71 (0.44–1.15)	0.58 (0.22–1.49)	0.54 (0.21–1.41)	0.57 (0.22–1.47)	0.58 (0.22–1.50)	0.58 (0.23–1.45)	0.61 (0.25–1.51)	0.68 (0.28–1.66)
Material GP fast: time effect								
Reference: 1 year	1	1	1	1	1	1	1	1
2 years	1.98 (1.74–2.24)	3.51 (2.73–4.53)	3.58 (2.77–4.61)	3.58 (2.78–4.62)	3.56 (2.77–4.59)	3.51 (2.73–4.51)	3.51 (2.74–4.51)	3.55 (2.76–4.56)
3 years	3.91 (3.04–5.03)	12.3 (7.44–20.5)	12.8 (7.69–21.3)	12.8 (7.72–21.4)	12.7 (7.65–21.1)	12.3 (7.48–20.3)	12.3 (7.49–20.3)	12.6 (7.62–20.8)
4 years	7.73 (5.31–11.3)	43.4 (20.3–92.7)	45.7 (21.3–98.0)	46.0 (21.5–98.7)	45.3 (21.1–97.0)	43.3 (20.5–91.5)	43.4 (20.5–91.7)	44.7 (21.0–94.8)
Material Equia: time effect								
Reference: 1 year	1	1	1	1	1	1	1	1
2 years	1.94 (1.69–2.23)	3.18 (2.45–4.12)	3.24 (2.49–4.22)	3.24 (2.49–4.21)	3.22 (2.48–4.18)	3.33 (2.57–4.33)	3.29 (2.54–4.27)	3.29 (2.54–4.26)
3 years	3.76 (2.85–4.96)	10.1 (6.02–17.0)	10.5 (6.22–17.8)	10.5 (6.20–17.7)	10.4 (6.15–17.4)	11.1 (6.59–18.8)	10.8 (6.44–18.2)	10.8 (6.45–18.1)
4 years	7.29 (4.81–11.0)	32.2 (14.8–69.9)	34.1 (15.5–74.9)	33.9 (15.4–74.4)	33.3 (15.2–72.8)	37.1 (16.9–81.3)	35.6 (16.4–77.6)	35.6 (16.4–77.3)
Upper jaw (ref: lower jaw)	---	---	---	---	0.96 (0.49–1.88)	---	0.78 (0.42–1.46)	0.75 (0.41–1.38)
Tooth type (reference: 4)								
5	---	---	---	---	3.42 (1.20–9.70)	---	3.23 (1.24–8.38)	3.11 (1.22–7.92)
6	---	---	---	---	2.02 (0.72–5.70)	---	2.72 (1.03–7.18)	2.43 (0.93–6.32)
7	---	---	---	---	1.40 (0.50–3.94)	---	4.06 (1.49–11.1)	3.32 (1.24–8.89)
8	---	---	---	---	1.20 (0.16–9.06)	---	6.09 (0.84–44.2)	6.79 (0.98–47.4)
Class (reference: class I)								
II	---	---	---	---	---	22.1 (9.53–51.2)	29.5 (12.0–72.6)	25.4 (10.4–62.0)
II MOD	---	---	---	---	---	94.3 (23.1–384)	102 (24.7–418)	95.7 (22.9–392)
Random part:								
Filling: intercept	---	3.18 (2.57–3.78)	1.78 (1.19–2.36)	1.80 (1.21–2.38)	1.78 (1.19–2.38)	1.58 (0.97–2.18)	1.56 (0.96–2.17)	1.52 (0.92–2.11)
Patient: intercept	---	---	2.70 (2.07–3.33)	2.65 (2.02–3.28)	2.59 (1.97–3.22)	2.26 (1.68–2.84)	2.17 (1.59–2.75)	1.82 (1.22–2.41)
Dentist: intercept	---	---	---	---	---	---	---	1.29 (0.75–1.83)

*interaction effect

**Table 7:** Random intercept models adjusted for fillings, patients, and dentists for all fillings (only within the manufacturer’s indications) with 0 year: including observations in the first half year (total = 1644 observations)

	Model 1	Model 2	Model 3	Model 4	Model 5	Model 6	Model 7	Model 8
				Adjusted for age and sex	Adjusted for age and sex	Adjusted for age and sex	Adjusted for age and sex	Adjusted for age and sex
*P* value for treatment	0.035	0.118	0.091	0.105	0.130	0.235	0.317	0.492
*P* value for treatment * time	0.845	0.686	0.747	0.728	0.678	0.853	0.743	0.762
Joint *P* value for treatment and treatment * time	<0.001	0.030	0.031	0.037	0.044	0.176	0.208	0.406
Fixed part: odds ratios								
Material Equia (reference: GP fast)	0.51 (0.27–0.95)	0.39 (0.12–1.27)	0.36 (0.11–1.18)	0.38 (0.12–1.23)	0.40 (0.12–1.31)	0.50 (0.16–1.57)	0.56 (0.18–1.74)	0.68 (0.23–2.04)
Material GP fast: time effect								
Reference: 1 year	1	1	1	1	1	1	1	1
2 years	1.88 (1.62–2.18)	3.27 (2.42–4.41)	3.29 (2.43–4.44)	3.30 (2.44–4.46)	3.30 (2.44–4.47)	3.30 (2.45–4.46)	3.31 (2.45–4.46)	3.30 (2.45–4.46)
3 years	3.52 (2.61–4.75)	10.7 (5.85–19.5)	10.8 (5.91–19.7)	10.9 (5.96–19.9)	10.9 (5.96–20.0)	10.9 (5.99–19.9)	10.9 (6.00–19.9)	10.9 (6.00–19.8)
4 years	6.61 (4.22–10.4)	34.9 (14.1–86.0)	35.5 (14.4–87.5)	35.9 (14.5–88.8)	36.0 (14.6–89.1)	36.1 (14.7–88.7)	36.2 (14.7–88.9)	36.1 (14.7–88.4)
Material Equia: time effect								
Reference: 1 year	1	1	1	1	1	1	1	1
2 years	1.92 (1.60–2.30)	3.01 (2.19–4.14)	3.08 (2.23–4.25)	3.08 (2.23–4.24)	3.04 (2.21–4.18)	3.18 (2.31–4.39)	3.10 (2.26–4.25)	3.11 (2.27–4.26)
3 years	3.69 (2.57–5.30)	9.09 (4.81–17.2)	9.48 (4.98–18.0)	9.47 (4.98–18.0)	9.23 (4.88–17.5)	10.1 (5.33–19.3)	9.59 (5.09–18.1)	9.69 (5.17–18.2)
4 years	7.09 (4.12–12.2)	27.4 (10.5–71.1)	29.2 (11.1–76.6)	29.2 (11.1–76.4)	28.1 (10.8–73.0)	32.3 (12.3–84.6)	29.7 (11.5–76.8)	30.2 (11.7–77.6)
Upper jaw (ref: lower jaw)	---	---	---	---	1.07 (0.46–2.49)	---	0.90 (0.40–1.99)	0.80 (0.37–1.72)
Tooth type (reference: 4)								
5	---	---	---	---	2.87 (0.86–9.52)	---	3.81 (1.22–11.9)	3.40 (1.13–10.2)
6	---	---	---	---	2.04 (0.58–7.09)	---	4.17 (1.25–13.9)	3.40 (1.06–11.0)
7	---	---	---	---	1.76 (0.51–6.12)	---	9.04 (2.48–33.0)	7.06 (2.04–24.5)
8	---	---	---	---	1.22 (0.07–20.0)	---	19.9 (1.30–304)	19.1 (1.38–264)
Class (reference: class I)								
Class II	---	---	---	---	---	24.3 (8.43–70.0)	45.0 (13.7–148)	38.7 (12.1–124)
Random part:								
Filling: intercept	---	3.15 (2.40–3.89)	1.80 (1.05–2.55)	1.82 (1.06–2.57)	1.81 (1.04–2.58)	1.82 (1.04–2.60)	1.79 (1.00–2.57)	1.76 (0.99–2.52)
Patient: intercept	---	---	2.55 (1.78–3.32)	2.53 (1.75–3.30)	2.46 (1.69–3.23)	2.07 (1.31–2.83)	1.92 (1.16–2.69)	1.23 (0.24–2.22)
Dentist: intercept	---	---	---	---	---	---	---	1.49 (0.86–2.12)

*interaction effect

## Discussion

Most of the previous research performed on GIC was carried out in either dental schools or other academic institutions as single-center studies; these studies are more prone to bias and may have lower methodological quality than multi-center studies [27, 28]. To obtain results that reflect the performance of GIC fillings in the real world, it is necessary to include dental offices with their daily practice time constraints and financially influenced treatment options in clinical trials [29]. Moreover, previous literature pointed out the advantages of including larger cohorts with their demographic characteristics in multi-center studies, which might influence the results [28–30], whereas clinical trials conducted in single centers often report results which are not the same as, and are frequently better than, those carried out in the field [29].

To the best of our knowledge, this is the first clinical trial that compared the high-viscosity Fuji IX GP® fast with an LC coating and Equia Fil® with a nanofilled resin coating. In addition, this is the first clinical trial that measured the clinical performance of the aforementioned materials within different statistical models considering effects related to patients and dentists on the FDI criteria [25]. The trial was conducted in 29 cities across Germany and incorporated 144 private dental clinics in order to evaluate both GIC materials under field conditions.

The clinical performance of both studied GIC materials was evaluated on permanent posterior teeth in both class I and class II (two and three surfaces) cavities of adult patients aged between 20 to 80 years old. The clinical performance of the tested materials was determined by evaluating the following aesthetic, functional, and biological criteria: surface luster, fracture and retention, marginal adaptation, occlusal wear, proximal contact and adaptation, patients view, postoperative sensitivity, caries recurrence, and tooth integrity.

Both materials showed similar good overall performance in class I cavities; however, in class II fillings, the Equia Fil® system with a nanofilled resin coating showed better overall performance with fewer failures in all the follow-up intervals. This is probably due to the nanofilled resin coating, which allows an improved primary stabilization of the filling material during the curing stage and improved infiltration and closure of the superficial defects within the GIC [31]. Nonetheless, the percentage of unsatisfactory to poor fillings according to FDI criteria was relatively high in two-surface class II fillings and higher in three-surface class II fillings (MOD) in both materials, with chippings and fractures being the main reason for the low scores. The reason can be attributed to the low flexural strength of GICs in general, which is a problem in cavities and not in single-surface fillings.

Cavity size in relation to the remaining tooth structure is a less considered criterion in most of the previous studies. The manufacturer indicates that Equia Fil® and other GIC materials can be used as permanent filling materials in cavities where the isthmus width is less than half the intercuspal distance. This recommendation was unexpectedly exceeded during placement by some of the dentists. Fillings that were placed within the manufacturers recommendation (*n* = 503) showed fewer failures and fewer poor to unsatisfactory scores than fillings that did not correspond entirely to the manufacturer’s recommendation. The difference in overall scores in relation to the manufacturer’s recommendation is evident in class II cavities and in three-surface cavities that are outside the manufacturer’s recommendations (see Tables 4 and 5).

Previous reports on GIC as a permanent filling material were mainly carried out in primary molars and within the atraumatic restorative treatment (ART) technique [16, 31, 32]. Studies on the clinical performance of Equia Fil® in occlusal cavities in permanent molars were either retrospective studies which reported good results when Equia Fil® was applied in class I and average class II cavities [19] or prospective trials which reported high success rates with Equia Fil® filling in class II cavities, with no influence of the cavity size on the performance of the filling [18]. The results of this study do not fully agree with the recent reports from Gurgan et al. [18]. While Gurgan et al. reported a 100 % success rate for Equia fillings in class I cavities and about 7 % (*n* = 2) marginal fractures in class II fillings, in our study, fractures in class I and class II fillings were reported within the first 2 years and in the third and fourth years. Additionally, cavity size was shown to be a determinant factor in the performance of the Equia filling, where fillings in class I cavities performed prominently better than those in class II fillings, and fillings with bigger cavities (MOD class II and cavities with large isthmus width) showed the highest fractures. Those findings are in line with the reports from Hickel et al. [16] and Frankenberger et al. [17], where coated GIC fillings did not perform as well in class II cavities as in class I, with fractures representing the main reason for failure in class II restorations.

Furthermore, the observed failures in this study can be distinguished into two different groups according to the FDI criteria: relative failures (the filling is evaluated as clinically unsatisfactory but repairable) and absolute failures (clinically poor), where monitored events require replacement of the filling. In the case of a relative failure (minor chipping of the filling), the filling was repaired and not replaced, whereas in the case of an absolute failure (fracture or loss of contact with adjacent tooth), the filling was replaced. Note that in this study we included both events for the evaluation score of fillings, while previous reports focused only on the total failure of the filling with fractures. Furthermore, in order to evaluate the performance of both materials and understand the influences behind the failures (categories 4 and 5), we included different statistical models adjusted for sex and age, each model measuring an additional influence on the performance of both materials (time effect, jaw, tooth location, cavity size, fillings per patient, and fillings by dentist). Moreover, we repeatedly calculated each model for (1) fillings within the manufacturer’s indications, (2) fillings within and without the manufacturer’s indications, and (3) all fillings including fillings missing at follow-ups (Tables 4 and 5). By comparing the observed outcomes between those different models, we noted remarkably fewer adverse observations (scores 4 and 5) when the fillings were limited to class I and conservative class II cavities. Fillings in large cavities (isthmus width larger than half the intercuspal distance) and three-surface fillings showed more adverse observations. This observation was also confirmed when evaluating both materials only for fractures and loss of retention (B5 criterion) and for loss of anatomical form (B8 criterion, only class II and class II MOD); see Tables 2 and 3.

A major challenge in this clinical trial was implementing a statistical model that is able to give a wide prospective evaluation of both materials based on the FDI evaluation criteria and scores, not only based on the failure of the filling material. Interpreting the results based only on survival estimation (e.g., the Kaplan-Meier estimate) would not reflect the true clinical performance of the materials tested, especially in a clinical trial in the field, where a multi-score evaluation scale is used to evaluate the tested materials. In addition, dropouts and irregular follow-ups of some of the patients would make it difficult to interpret the results using only survival estimation.

## Conclusion

Within the limitations of the study, we can conclude that no significant difference in performance between both materials was found within 4 years. However, Equia Fil® with a nanofilled resin coating showed a slightly better overall performance than the conventional Fuji IX GP® fast with the LC coating and an overall lower odds to failure. Both materials performed well in class I cavities. In class II cavities, the dentist must pay attention to the cavity size. It was shown that higher odds of failure are associated with class II cavities, especially in large cavities and three-surface fillings (i.e., MOD class II), which indicate that the manufacturer’s recommendations have to be followed.

## Additional files

Additional file 1 Additional tables. (DOCX 20 kb) Additional file 2 CONSORT checklist. (DOC 324 kb) Additional file 3 CONSORT flow diagram. (DOC 285 kb)

## Abbreviations

ART: atraumatic restorative treatment
DSMC: Data Safety and Monitoring Committee
GCP: Good Clinical Practice
GEP: Good Epidemiological Practice
GIC: glass ionomer cement
GMP: Good Manufacturing Practices
MOD: mesial-occlusal-distal surfaces
RCT: randomized clinical trial
SCS: single-center studies
SES: socio-economic status
USPHS: United States Public Health Service
