# Supplementary material for: Clinical performance during 48 months of two current glass ionomer restorative systems with coatings: a randomized clinical trial in the field
Source: Trials. 2016 May 8;17:239. doi: 10.1186/s13063-016-1339-8 (PMC4860258; doi:10.1186/s13063-016-1339-8)
Supplement: Additional file 1 — Additional tables. (DOCX 20 kb) [file 13063_2016_1339_MOESM1_ESM.docx]

Explanation of the high odds ratios:

The last three columns of eTable 1 show the number of cases and non-cases used in the main analyses. The eTable 2 shows the cell numbers for the odds ratios of model I in eTable 3: class II filling: 266*643/38/686 = 6.56; class III fillings: 36*643/38/44 = 13.8. In this model, however, the 1713 observations are assumed to be independent, which is not justified. Even the model II is not well justified because different fillings of the same patient are assumed to be independent. Only model III is valid with respect to the assumptions of independence of residuals. But this model III in eTable 3 shows high odds ratios, similar to those in model II. Obviously, the high odds ratios are rooted in accounting for the filling level (with up to 4 time observations each).

eTable 1: The **overall** clinical performance score (number and percentage) for both materials (n=782) in 510 patients with 1713 follow-up exams

|  | 1 year | | 2 years | | 3 years | | 4 years | | total | Case? | Total |
| --- | --- | --- | --- | --- | --- | --- | --- | --- | --- | --- | --- |
| Material | GPfast | Equia | GPfast | Equia | GPfast | Equia | GPfast | Equia |  |  |  |
| Score 1  (clinically excellent) | 37 (13) | 53 (16) | 30 (13) | 44 (16) | 22 (12) | 20 (12) | 10 (8) | 18 (15) | 234 | No | 1373 |
| Score 2  (clinically good) | 137 (46) | 150 (46) | 108 (46) | 151 (54) | 79 (43) | 77 (47) | 49 (41) | 52 (44) | 803 | No |  |
| Score 3  (clinically sufficient) | 71 (24) | 78 (24) | 47 (20) | 53 (19) | 33 (18) | 26 (16) | 13 (11) | 15 (13) | 336 | No |  |
| Score 4  (clinically unsatisfactory) | 40 (13) | 32 (10) | 27 (12) | 12 (4) | 13 (7) | 13 (8) | 8 (7) | 2 (2) | 147 | Yes | 340 |
| Score 5  (clinically poor) | 10 (3) | 10 (3) | 22 (9) | 18 (6) | 35 (19) | 28 (17) | 38 (32) | 32 (27) | 193 | yes |  |
| Total | 295 | 323 | 234 | 278 | 182 | 164 | 118 | 119 | 1713 |  | 1713 |

eTable 2: Number of cases and non-cases for both materials in categories of filling class (n=782) in 510 patients with 1713 follow-up exams

| Class | Cases  (clinically unsatisfactory or poor) | Non-cases (clinically sufficient or better) | Total |
| --- | --- | --- | --- |
| I | 38 | 643 | 681 |
| II | 266 | 686 | 952 |
| III | 36 | 44 | 80 |
| Total | 340 | 1373 | 1713 |

eTable 3: Random intercept models of filling class (without 0 year: 1713 observations on 782 fillings and 510 subjects and 101 dentists)

|  | Model I | Model II | Model III | Model IV | Model 6 as  in table xx |
| --- | --- | --- | --- | --- | --- |
|  |  |  |  |  |  |
| Class (reference: F1) |  |  |  |  |  |
| F2 | 6.56  (4.59 – 9.37) | 37.4 (13.6 - 103) | 33.9 (12.2 – 94.3) | 32.5 (11.7 – 90.1) | 42.6 (13.9 - 131) |
| F3 | 13.8 (8.00 – 24.0) | 195 (31.8 - 1196) | 205 (35.4 - 1191) | 179 (30.7 - 1046) | 296 (43.5 - 2013) |
| **Adjusted for** |  |  |  |  |  |
| **Fixed part:** |  |  |  |  |  |
| Age | No | No | No | Yes | Yes |
| Sex | No | No | No | Yes | Yes |
| Treatment | No | No | No | No | Yes |
| Time | No | No | No | No | Yes |
| Treatment*time | No | No | No | No | Yes |
|  |  |  |  |  |  |
| **Random part:** |  |  |  |  |  |
| Filling: Intercept | No | yes | yes | yes | yes |
| Patient: Intercept | No | No | yes | yes | yes |

Table 2: **First part** of the description of the **overall** clinical performance score: Number and percentage of fractured fillings in distributed follow-up checks

Table 3: **Second part** of the description of the **overall** clinical performance score: Number and percentage of 2 and 3 surface fillings that lost approximal contact in distributed follow-up checks
