# Supplementary material for: Clinical performance during 48 months of two current glass ionomer restorative systems with coatings: a randomized clinical trial in the field
Source: Trials. 2016 May 8;17:239. doi: 10.1186/s13063-016-1339-8 (PMC4860258; doi:10.1186/s13063-016-1339-8)
Supplement: Additional file 3 — CONSORT flow diagram. (DOC 285 kb) [file 13063_2016_1339_MOESM3_ESM.doc]

**
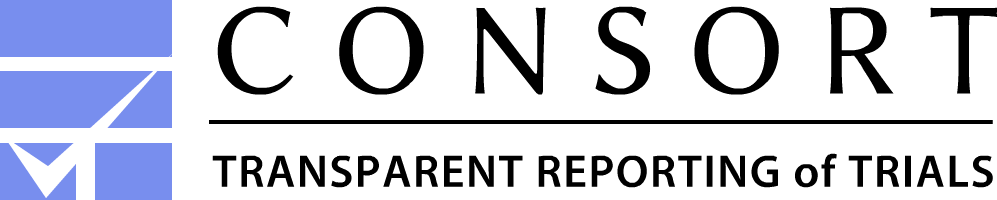
**

**CONSORT 2010 Flow Diagram**

**Enrollment**

**Allocation**

Fillings assessed for eligibility (n = 1006)

Excluded (n = 5 )

♦  Not meeting inclusion criteria (n = 5 )

Allocated to intervention (study arm EQUIA,
n = 515)

♦ Received allocated intervention (n = 398 )

♦ Did not receive allocated intervention (outside manufacturer’s instruction)
(n = 131)

Randomized (n = 1001)

Allocated to intervention (study arm FUJI IX,
n = 486)

♦ Received allocated intervention (n = 384)

♦ Did not receive allocated intervention (outside manufacturer’s instruction)
(n = 130)

**Follow-Up 1-4**

Follow-up observations (n = 860)
Follow-up incomplete (due to total dropouts) (n = 376)
Intervention canceled (reasons):
Study termination/Lost in follow-up (n = 343)
Patient / Dental office moved (n = 33)

Follow-up observations (n = 925)
Follow-up incomplete (due to total dropouts) (n = 401)
Intervention canceled (reasons):
Study termination/Lost in follow-up (n = 364)
Patient / Dental office moved (n = 37)

**Analysis**

Analysed examinations (n = 700)
♦ Excluded from analysis (out of manufacturers instruct., (n = 27))

Analysed examinations (n = 793)
♦ Excluded from analysis (out of manufacturers instruct.,(n = 33))
